# Illness perceptions are associated with mortality among 1552 colorectal cancer survivors: a study from the population-based PROFILES registry

**DOI:** 10.1007/s11764-016-0536-5

**Published:** 2016-03-19

**Authors:** Melissa S. Y. Thong, Adrian A. Kaptein, Pauline A. J. Vissers, Gerard Vreugdenhil, Lonneke V. van de Poll-Franse

**Affiliations:** 1 Department of Medical Psychology, Academic Medical Center University of Amsterdam, P.O. Box 22660, 1100 DD Amsterdam, Netherlands; 2 Department of Medical Psychology, Leiden University Medical Center, Leiden, Netherlands; 3 Netherlands Comprehensive Cancer Organisation (IKNL), Utrecht, Netherlands; 4 Department of Internal Medicine, Máxima Medical Centre, Veldhoven, Netherlands; 5 Department of Medical Oncology, Maastricht University Medical Centre, Maastricht, Netherlands; 6 Division of Psychosocial Research and Epidemiology, The Netherlands Cancer Institute, Amsterdam, Netherlands

**Keywords:** Cancer, Illness perceptions, Mortality, Population-based, Survivors

## Abstract

**Purpose:**

Cancer survivors construct perceptions of illness as a (mal)adaptive mechanism. These perceptions motivate/drive subsequent self-management behaviors toward symptoms and treatment that influence health outcomes. Negative illness perceptions have been associated with increased mortality in other chronically ill groups. However, this association is under-researched in cancer survivors. We aimed to explore the association between illness perceptions and mortality in stage I–III progression-free colorectal cancer (CRC) survivors.

**Methods:**

We used data from the population-based Patient Reported Outcomes Following Initial treatment and Long Term Evaluation of Survivorship (PROFILES) registry of two CRC survivorship studies conducted in 2009 and 2010. We accessed clinical data from the Netherlands Cancer Registry, and mortality data from municipal personal records database. Follow-up was until 31 December 2014. Survivors (*n* = 1552) completed the Brief Illness Perception Questionnaire. Cox proportional hazard models estimated the association between illness perceptions and mortality.

**Results:**

Negative illness perceptions on consequences (adjusted hazard ratio (HR_adj_) 1.60, 95 % confidence interval (CI) 1.14–2.25) and emotion (HR_adj_ 1.65, 95 % CI 1.18–2.31) were associated with higher mortality, after adjusting for demographic, clinical, and lifestyle factors. Smoking and inadequate physical activity were independently associated with mortality for all Brief Illness Perception Questionnaire (BIPQ) dimensions.

**Conclusions:**

Survivors’ perceptions of their illness are important as these perceptions may influence health outcomes during survivorship period. Clinical practice needs to identify and address maladaptive illness perceptions to support more adaptive self-management behaviors and enhance survivorship.

**Implications for cancer survivors:**

Cancer survivors may benefit from interventions that address potentially maladaptive perceptions and encourage more adaptive self-management behaviors.

## Introduction

Cancer survivors with similar clinical characteristics can have vastly different perceptions of and responses to their illness. These differences, postulated by the Common Sense Model (CSM) of self-regulation, are due to the personal model or representation of illness that individuals construct as a (mal)adaptive mechanism when confronted with an illness [1]. Representations, cognitive and emotional, can be informed by previous experience, observations, or received information of the illness and related symptoms [2]. These representations or illness perceptions motivate subsequent self-management behaviors such as lifestyle changes [3, 4] and treatment adherence [5] that can determine health outcomes. The CSM is a relevant model for cancer survivorship as it considers individuals as problem solvers actively involved in the management of their own health. It implies that maladaptive cognitions of cancer survivors can be addressed, through interventions, to achieve better health outcomes when they have a more adaptive understanding of their condition and are able to evaluate the effects of acting on this understanding [6].

Among cancer survivors, negative illness perceptions have been associated with poorer self-efficacy to manage cancer- or treatment-related problems [7], and poorer self-management behaviors such as passive or maladaptive coping [8, 9], and poorer treatment adherence [10]. Individuals with negative perceptions also report poorer health outcomes such as higher symptom burden [11], psychological distress [12], and lower quality of life [13]. The association between illness perceptions and mortality has been studied in other chronic diseases such as end-stage renal disease [14] and heart disease [15], whereby negative illness perceptions were associated with higher mortality risks. Research into the association between illness perceptions and mortality among cancer survivors is scarce. In a study of newly diagnosed breast and non-metastatic colorectal cancer (CRC) survivors, not believing in the curability of cancer has been associated with increased mortality during 15 years of follow-up [16, 17].

Understanding the association of illness perceptions with mortality among cancer survivors is especially relevant as these individuals are living longer due to earlier detection and improved treatments [18]. This trend is also noted in CRC where survival has improved in recent years [19, 20]. CRC is the third most common cancer, with an annual number of new cases of approximately 132,000 in the USA [21] and 342,000 in Europe [22]. In the Netherlands, the number of individuals surviving CRC is projected to increase from 58,000 in 2009 to approximately 92,000 by 2020, of which >50 % are considered long-term survivors (≥5 years post-diagnosis) [23]. This rapidly growing group is expected to self-manage late- and long-term effects of CRC and its treatment whose self-management strategies may be influenced by illness perception [24]. Emerging results indicate a link between self-management activities on lifestyle factors and CRC survival [25, 26].

Our study aimed to explore the association of illness perceptions with mortality in progression-free CRC survivors diagnosed within 5 years of survey and treated with curative intent. We hypothesize that CRC survivors with more negative illness perceptions will have higher mortality risk compared with survivors with more positive illness perceptions.

## Methods

### Setting and participants

For this study, we included individuals diagnosed with stage I–III CRC between 2004 and 2009 in South Netherlands as registered in the Netherlands Cancer Registry (NCR). The NCR records data on all individuals who are newly diagnosed with cancer in the southern part of the Netherlands. Exclusion criteria included cognitive impairment, had died prior to start of the study (according to the Central Bureau for Genealogy which collects information on all deceased Dutch citizens via the civil municipal registries, and hospital records) or had unverifiable addresses. For a complete overview of sample selection process, please refer to the Patient Reported Outcomes Following Initial treatment and Long Term Evaluation of Survivorship (PROFILES) registry website under “data & documentation” (http://www.profilesregistry.nl/dataarchive/study_units/view/22).

Ethical approval for the study was obtained from a local certified Medical Ethics Committee of the Maxima Medical Centre Veldhoven, Netherlands.

### Data collection

This secondary study pooled data from two separate surveys on CRC survivors conducted in January 2009 and December 2010 to evaluate patient-reported outcomes. Details of these studies are reported elsewhere [27]. Data collection was completed within PROFILES registry as previously described [28]. In short, eligible survivors invited to participate in the study were given the option of completing either an online or paper questionnaire. Survivors were reassured that non-participation had no consequences on their follow-up care or treatment. All participants provided informed consent. Non-respondents were sent a reminder letter and questionnaire within 2 months. Data from PROFILES is linked directly to clinical data from the NCR and is readily available for research purposes from PROFILES (www.profilesregistry.nl) [28].

### Study measures

#### Illness perceptions

Respondents completed the Dutch version of the Brief Illness Perception Questionnaire (BIPQ) [29] at time of survey. The BIPQ has sound psychometric properties and has been used with cancer populations (www.uib.no/ipq) [30]. The BIPQ consists of eight items: Five items assess cognitive representations (consequences, timeline, personal control, treatment control, identity), two items assess emotional representations (concern, emotion), and one item assesses illness comprehensibility (coherence). It uses a single-item scale approach to assess perceptions on a linear 1–10 point scale. The responses of three items (personal control, treatment control, coherence) were recorded to be in the same direction as the other items for statistical analyses. Higher scores indicated more negative perceptions.

#### Demographics, clinical, and lifestyle data

Self-reported demographic data included marital status and education level. Comorbid status at the time of survey was categorized according to the adapted Self-Administered Comorbidity Questionnaire (SCQ) [31].

Survivors’ demographics and clinical information including date of birth, date of diagnosis, clinical stage according to the tumor–node–metastasis clinical classification [32], and treatment were accessed from the NCR.

Lifestyle factors included self-reported smoking, alcohol use, body mass index (BMI), and physical activity. BMI was calculated with self-reported height and weight. On the validated European Prospective Investigation into Cancer (EPIC) Physical Activity Questionnaire [33], survivors reported the average time spent, during winter and summer, on walking, cycling, gardening, household activities, and sports. Hours per week spent on moderate-to-vigorous physical activity (MVPA) were derived from estimated metabolic equivalent intensity values assigned to each activity based on previously described classifications [34, 35].

#### Disease progression

Data of metachronous metastases occurring between initial cancer diagnosis and time of survey were retrospectively retrieved from medical files [36].

### Statistical analyses

We had previously found that CRC survivors with metastatic disease had significantly more negative illness perceptions than survivors with non-metastatic disease [37]. Therefore, to assess the relative risk of illness perceptions and mortality, we included only survivors with no metachronous metastasis at time of survey in our analyses. To facilitate interpretation of results in daily clinical practice and to identify the survivors with the most negative illness perception, we dichotomized the BIPQ scores using the 75th interquartile range score as cutoff, as previously done [38].

To determine the prevalence of survivors who scored negatively on multiple BIPQ dimensions, we derived two scale scores from the dichotomized BIPQ scores: cognitive and emotional representation. We summed the BIPQ dimensions categorized as negative for each subscale: cognitive representation (consequences, timeline, personal control, treatment control, identity) and emotional representation (concern, emotion). Differences in demographic and clinical characteristics, and lifestyle factors between respondents with negative (e.g., the top 10 % of patients) and positive representations were assessed using independent sample *t* test or Mann–Whitney test, and chi-square test, where appropriate.

Cox proportional hazard models were used to estimate the associations between BIPQ dimensions and mortality. Confounders for adjustment in the Cox models were selected a priori. These included age at survey, sex, education, marital status, comorbidity, cancer type, cancer stage, smoking, alcohol consumption, and physical activity. As survivors were assessed 1–5 years after cancer diagnosis, the Cox models were left-truncated and the time of questionnaire completion was set as entry time to minimize survivorship bias. Time since cancer diagnosis was used as the underlying time scale for the Cox regression. Follow-up time was calculated from cancer diagnosis until death or end of follow-up on 31 Dec 2014, whichever occurred first.

All tests were two-sided and significant if *p* < 0.05. Descriptive analyses were performed using Statistical Package for Social Sciences (SPSS, Chicago, IL, USA) version 22 for Windows, and Cox regression analyses were performed using SAS statistics version 9.3 (SAS Institute, Inc., Cary, NC).

## Results

### Survivors’ characteristics

In total, 2229 (70 %) survivors responded. Differences in characteristics among respondents, non-respondents, and patients with non-verifiable addresses have been reported elsewhere [27]. Of the respondents, 6 had emigrated before end of follow-up, 9 had survey dates that occurred before start of study, 457 were not reviewed for metachronous metastases, 135 had metachronous metastasis at time of survey, and 70 did not complete any BIPQ dimensions. These respondents were excluded in the analyses, resulting in a final sample of 1552 survivors.

### Illness perceptions

The median and 75th interquartile BIPQ scores for the sample are shown in Table 1. The percentage of survivors who scored negatively on the BIPQ dimensions ranged between 20 and 23 %.

**Table 1:** Brief Illness Perception Questionnaire (BIPQ) dimension scores of stage I–III progression-free CRC survivors (*n* = 1552)

BIPQ dimension	Description	Missing values	Median (75th IQS)	*n* (%) of patients with negative BIPQ score^a^
BIPQ1: consequences	How much does your illness affect your life? (1: no affect; 10: severely affects)	8	3 (6)	311 (20)
BIPQ2: timeline	How long do you think your illness will continue? (1: very short time; 10: forever)	81	3 (7)	325 (21)
BIPQ3: personal control	How much control do you feel you have over your illness? (1: extreme control; 10: absolutely no control)	53	6 (9)	338 (22)
BIPQ4: treatment control	How much do you think your treatment can help your illness? (1: extremely; 10: not at all)	95	4 (5)	327 (21)
BIPQ5: identity	How much do you experience symptoms from your illness? (1: no symptoms at all; 10: many severe symptoms)	40	3 (5)	351 (23)
BIPQ6: concern	How concerned are you about your illness? (1: not concerned; 10: extremely concerned)	30	4 (6)	313 (20)
BIPQ7: coherence	How well do you feel you understand your illness? (1: understand clearly; 10: do not understand)	46	3 (6)	313 (20)
BIPQ8: emotion	How much does your illness affect you emotionally? (1: not at all; 10: extremely)	36	3 (5)	338 (22)

^a^The 75th interquartile score (IQS) was used as the cutoff, whereby higher score indicates negative perceptions

On the prevalence of survivors with negative perceptions on multiple BIPQ items, 12 % scored negatively on at least three of the five cognitive BIPQ dimensions and 13 % scored negatively on both emotional BIPQ dimensions. Survivors with negative cognitive representations were more likely to have rectal cancer, treated with surgery and radiotherapy, and have high education level, and less likely to meet the Dutch physical activity guidelines (Table 2). For negative emotional representations, significant differences were found for time since diagnosis and BMI, whereby shorter time since diagnosis and higher BMI were associated with negative emotional representations. Survivors with negative cognitive or emotional representations were more likely to have two or more comorbid conditions than survivors with positive representations. However, significant differences in prevalence of heart conditions, diabetes mellitus, and osteoarthritis were noted only among survivors with negative cognitive representations. Current drinkers were more likely to have positive cognitive or emotional representations.

**Table 2:** Demographic and clinical characteristics of stage I–III progression-free CRC survivors (*n* = 1552), stratified by cognitive and emotional representations as measured with the Brief Illness Perception Questionnaire (BIPQ)

*n* (%)	Cognitive representation scale^a^			Emotional representation scale^b^		
	Negative (*n* = 185)	Positive (*n* = 1367)	*p* value	Negative (*n* =195)	Positive (*n* =1357)	*p* value
Male	106 (57)	772 (57)	0.8	102 (52)	776 (57)	0.2
Mean age at survey + SD	68.4 ± 10.5	69.6 ± 9.4	0.1	68.1 ± 9.6	69.6 ± 9.6	0.03
Mean years since initial diagnosis ± SD	2.9 ± 0.9	2.9 ± 1.0	0.5	2.8 ± 1.0	2.9 ± 1.0	0.02
Type of cancer			<.0001			0.1
Colon	97 (52)	923 (68)		118 (67)	902 (67)	
Rectal	88 (48)	444 (33)		77 (40)	455 (34)	
Cancer stage			0.8			0.8
I	61 (33)	424 (31)		60 (31)	425 (31)	
II	70 (38)	549 (40)		81 (42)	538 (40)	
III	54 (29)	394 (29)		54 (28)	394 (29)	
Primary treatment			<.0001			0.1
SU	75 (41)	731 (54)		90 (46)	716 (53)	
SU + RT	65 (35)	276 (20)		56 (29)	285 (21)	
SU + CT	31 (17)	288 (21)		39 (20)	280 (21)	
SU + RT + CT	14 (8)	72 (5)		10 (5)	76 (6)	
No. of self-reported comorbid conditions			<.0001			0.001
None	38 (21)	431 (32)		45 (23)	424 (31)	
One	37 (20)	399 (29)		45 (23)	391 (29)	
Two or more	110 (60)	537 (39)		105 (54)	542 (40)	
Prevalent comorbid conditions						
Heart condition	42 (26)	224 (19)	0.04	40 (24)	226 (19)	0.1
High blood pressure	75 (47)	429 (36)	0.01	70 (42)	434 (37)	0.1
Diabetes mellitus	37 (24)	179 (16)	0.01	29 (18)	187 (16)	0.5
Osteoarthritis	59 (38)	335 (29)	0.03	57 (35)	337 (30)	0.1
In partnered relationship	131 (71)	1033 (76)	0.1	144 (74)	1020 (75)	0.7
Socioeconomic status			0.6			0.3
Low	39 (22)	310 (23)		45 (24)	304 (23)	
Medium	70 (40)	535 (41)		83 (45)	522 (40)	
High	60 (35)	457 (35)		55 (30)	462 (35)	
Education level			0.03			0.7
Low	27 (15)	258 (19)		39 (20)	246 (19)	
Medium	103 (57)	809 (60)		117 (60)	795 (60)	
High	51 (28)	272 (20)		38 (20)	285 (22)	
Body mass index	27.2 ± 5.0	26.8 ± 4.3	0.3	27.6 ± 5.1	26.7 ± 4.3	0.03
Current smoker	27 (15)	149 (11)	0.1	25 (14)	151 (11)	0.3
Current alcohol drinker	85 (57)	734 (66)	0.02	83 (54)	736 (66)	0.003
Moderate-to-vigorous physical activity (hours/week)	8.6 ± 7.7	11.0 ± 8.9	<.0001	10.2 ± 8.5	10.8 ± 8.9	0.3

Percentages may not add up to 100 % due to rounding up of decimals

^a^Cognitive representation scale: consequences, timeline, personal control, treatment control, and identity. Negative cognitive representation: scored negatively in at least three of the five dichotomized cognitive BIPQ dimensions

^b^Emotional representation scale: concern and emotion. Negative emotional representation: scored negatively on two out of two dichotomized emotional BIPQ representations

Percentages may not add up to 100 % due to rounding up of decimals

### Illness perceptions and mortality

As of 31 December 2014, 249 (16 %) respondents had died. The mean time from survey completion to end of follow-up was 4.4 ± 1.3 years.

In the unadjusted Cox model, negative perceptions on consequences, timeline, identity, and emotion were associated with higher mortality risks (Table 3, model 1). Following full adjustment of demographic, clinical, and lifestyle variables (Table 3, model 4), negative perceptions on consequences (adjusted hazard ratio (HR_adj_) 1.60, 95 % confidence interval (CI) 1.14–2.25) and emotion (HR_adj_ 1.65, 95 % CI 1.18–2.31) remained significantly associated with higher mortality risks. Lifestyle factors such as smoking and physical activity were independently associated with mortality for all BIPQ dimensions. The adjusted hazard estimates of being a current smoker ranged between 2.32 and 2.46 (data not shown). Survivors who meet the Dutch guidelines for physical activity per week had approximately 4 % reduced risk of mortality (data not shown).

**Table 3:** Unadjusted and adjusted Cox proportional hazard risk estimates of illness perceptions on mortality of stage I–III progression-free CRC survivors (*n* = 1552)

BIPQ dimension	BIPQ score^a^	Deaths	Model 1 (unadjusted); HR (95 % CI)	Model 2 (Model 1 + demographics); HR (95 % CI)	Model 3 (Model 2 + clinical); HR (95 % CI)	Model 4 (Model 3 + lifestyle); HR (95 % CI)
BIPQ1 - consequences	+	184/1233	ref	ref	ref	ref
	−	64/311	1.47 (1.11-1.96)	1.66 (1.23-2.23)	1.62 (1.21-2.18)	1.60 (1.14-2.25)
BIPQ2 - timeline	+	166/1146	ref	ref	ref	ref
	−	69/325	1.57 (1.19-2.08)	1.43 (1.07-1.91)	1.44 (1.07-1.93)	1.32 (0.94-1.86)
BIPQ3 - personal control	+	185/1161	ref	ref	ref	ref
	−	54/338	0.96 (0.71-1.30)	0.91 (0.67-1.24)	0.93 (0.68-1.27)	1.00 (0.70-1.42)
BIPQ4 - treatment control	+	168/1135	ref	ref	ref	ref
	−	62/327	1.30 (0.97-1.74)	1.15 (0.85-1.55)	1.16 (0.86-1.57)	1.13 (0.80-1.58)
BIPQ5 - identity	+	172/1161	ref	ref	ref	ref
	−	68/351	1.34 (1.01-1.77)	1.54 (1.15-2.05)	1.51 (1.13-2.02)	1.31 (0.92-1.85)
BIPQ6 - concern	+	184/1209	ref	ref	ref	ref
	−	56/313	1.25 (0.93-1.69)	1.47 (1.09-2.00)	1.48 (1.09-2.01)	1.35 (0.94-1.92)
BIPQ7 - coherence	+	179/1193	ref	ref	ref	ref
	−	57/313	1.18 (0.88-1.59)	1.25 (0.93-1.69)	1.25 (0.92-1.69)	1.07 (0.75-1.52)
BIPQ8 – emotion	+	171/1178	ref	ref	ref	ref
	−	66/338	1.38 (1.04-1.84)	1.67 (1.25-2.24)	1.63 (1.22-2.19)	1.65 (1.18-2.31)

Demographics: age at survey, gender, relationship status, and education level; clinical: type of cancer, stage of cancer, and comorbid conditions; lifestyle: BMI, smoking, alcohol consumption, and physical activity

*HR* hazard ratio, *CI* confidence interval

^a^BIPQ: Brief Illness Perception Questionnaire. The 75th interquartile score was used as the cutoff, whereby higher score indicates negative perceptions

## Discussion

In this large population-based study of stage I–III progression-free CRC survivors, approximately one fifth of respondents had negative illness perceptions. Negative perceptions on consequences and emotions remained associated with higher risk of mortality, after adjusting for a range of demographic, clinical, and lifestyle variables. These results are in line with previous research which reported that newly diagnosed CRC survivors with negative belief in the curability of cancer had higher mortality risks in 15 years of follow-up [17].

In our study, negative perceptions on consequences and emotions remained significant predictors of mortality, even after extensive adjustments of possible confounders. Perceptions of more negative consequences has been shown to be one of the strongest predictors of poorer health outcomes and slower return to work in a range of chronic illness including cancer [30]. Cancer survivors on chemotherapy who attribute their symptom burden as negative consequences of their illness experienced more psychological distress [11]. Similarly, negative emotion was associated with higher mortality. Studies suggest that stress-related adaption processes could have physiological consequences such as alterations in cellular immune function and pro-inflammatory signaling during cancer survivorship which in turn could influence disease progression [39].

The strong association between illness perceptions and mortality risk among the chronically ill attests to the importance of cognitive and behavioural adaptation to chronic illness. Among cancer survivors, negative perceptions have been associated with poorer self-efficacy [7] and poorer self-management behaviors such as passive coping [8]. Pre-treatment negative illness perceptions have been associated with maladaptive coping such as venting, substance use, and denial in head and neck cancer survivors [9]. Negative perceptions about treatment efficacy can also influence adherence to treatment recommendations [10]. In our study, a significant proportion of survivors (21 %) perceived their treatment as being not helpful. These negative perceptions could influence subsequent self-management behaviors. We found that survivors with negative cognitive representations were physically less active, probably as they also had more comorbidity. Insufficient physical activity and being a current smoker were independent risk factors of mortality in all BIPQ dimensions (data not shown). This is in line with previous research of a link between lifestyle factors and CRC survival [25, 26]. These points taken together highlight the need for intervention to address possible maladaptive perceptions and improve survivors’ self-management strategies for a healthier survivorship and better clinical outcome. Such intervention is especially salient for CRC survivors as they tend to be older. Older cancer survivors have been found to be less aware of the benefits of lifestyle changes for better health outcomes [40].

Our results contribute to the growing body of research that negative illness perceptions are a significant risk factor for mortality that clinicians need to address [16, 17]. In contrast to other significant risk factors such as age, comorbidity, or cancer stage (data not shown), maladaptive illness perceptions have shown to be amenable to psycho-educational interventions in other cancer populations. A physical activity intervention to address shoulder morbidity among breast cancer survivors reduced perceptions of symptom severity and perceived consequences [41]. Positive changes in illness perceptions improved emotional well-being among breast cancer survivors who attended a psychosocial aftercare program [42]. A cognitive-behavioral stress management intervention targeting negative affect and cognitions was shown to downregulate anxiety-related pro-inflammatory and metastasis-related gene expression in circulating leukocytes in early stage breast cancer survivors [43].

Our study has limitations. The years from survey completion until end of follow-up is relatively short. Our sample consisted of a cross section of CRC survivors with varying years since initial CRC diagnosis. Therefore, it is plausible that shorter-term survivors, compared with longer-term survivors, report more negative illness perceptions. We addressed possible survivorship bias by using a left-truncated Cox regression model. We could not confirm the metastasis status for a proportion of respondents. Mortality estimates should be interpreted with caution as exclusion of these respondents could have biased the results. Although we have corrected for a range of factors, there is a possibility of residual confounding. For example, we did not explicitly assess self-management strategies. Nevertheless, our study is one of the first to assess the mortality risks associated with illness perceptions in a large population-based CRC sample with a high response rate.

In conclusion, our study adds to the growing body of research highlighting the importance of assessing survivors’ perceptions of their illness as these can influence health outcomes during survivorship period. Research shows that illness perceptions are amenable to intervention. It is therefore important to address potentially maladaptive perceptions and encourage more adaptive self-management behaviors among CRC survivors treated curatively to ensure that they achieve better health outcomes during survivorship.
